# *Mycobacterium shimoidei* cavitary pneumonia*:* A rare case report, literature review

**DOI:** 10.1016/j.jctube.2025.100530

**Published:** 2025-04-27

**Authors:** Siddartha Guru, David Ingram

**Affiliations:** Infectious Disease, Penn State Hershey Medical Center, Hershey, PA, USA

**Keywords:** *Mycobacterium shimoidei*, Case report, Non-tuberculous mycobacterium infections

## Abstract

*Mycobacterium shimoidei* is a rare non-tuberculous mycobacterium (NTM) which causes pneumonia. Since its discovery in 1975, less than 50 cases have been published and this would be only the fourth case in the US. We present a case of *Mycobacterium shimoidei* in a 72-year-old male with symptoms of cough, dyspnea, and weight loss with cavitary lung lesion on imaging. Sputum cultures grew *Mycobacterium shimoidei* in two separate collections, and the patient was treated with oral azithromycin, ethambutol, and rifabutin empirically. Though due to medication side effects rifabutin was stopped and other antibiotics were attempted based on susceptibilities but he was unable to tolerate any of them. Thus, only a two-drug regimen including ethambutol and azithromycin was used, on which he improved clinically and cavitary lung lesions decreased in size. In addition, we did a literature review and compiled 41 previously published cases of *Mycobacterium shimoidei.*

## Highlights

• Mycobacterium shimoidei is rare cause of non-tuberculous mycobacterium infections.
• Most common risk factors include COPD and previous Tuberculosis infection.
• Most isolates are susceptible to ethambutol, clarithromycin and rifabutin.
• A combination of 2–3 antibiotics used for duration of 12–18 months.

## 1 Introduction

Nontuberculous mycobacterium (NTM) infections have risen globally, including in the US, with prevalence increasing from 6.78 to 11.7 per 100,000 person-years between 2008 and 2015 [1,2]. Over 170 species and subspecies of nontuberculous mycobacterium (NTM) exist, but only some species cause human infections [3]. *Mycobacterium shimoidei* is an exceptionally rare NTM that predominantly causes pulmonary infections. We report the fourth known case of *Mycobacterium shimoidei* in the US, and less than 50 cases have been reported worldwide.

## 2 Case report

**Fig. 1:**
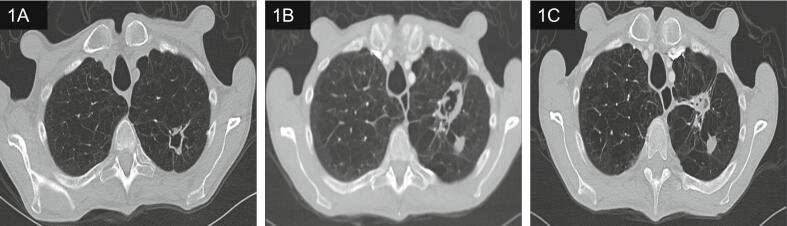
**(a).** CT chest shows an irregular cavitary lesion in the posterior left upper lobe measuring 3.18 mm × 18.7 mm with widespread small irregular noncalcified nodules in bilateral lung fields and moderate upper lobe predominant centrilobular emphysematous disease. (**b).** A new anterior left upper lobe cavitary lesion measuring 38.6 mm × 24.0 mm with the old posterior cavitary lesion with volume loss measuring 14 mm × 10.6 mm and the diffuse small noncalcified nodules. **(c).** Decrease in size of the anterior lesion measuring 28.7 mm × 14. 8 mm and stable posterior lesion measuring 13.5 mm × 11.9 mm.

A 72-year-old male with a history of chronic obstructive pulmonary disease (COPD) presented to the clinic with a worsening chronic cough and shortness of breath over the past six months with unintentional thirty-five pounds of weight loss over two years. Denied fevers, chills, night sweats, and any history or known contact with tuberculosis. He had a 43-pack-year smoking history. He served in the Navy and worked as a welder for 35 years. Family history was significant for lung cancer. He was initially treated with a course of azithromycin and prednisone for possible COPD exacerbation. Two months later, a computed tomography (CT) of the chest was obtained for persistent symptoms showed an irregular cavitary lesion in the posterior left upper lobe measuring 3.18 mm x 18.7 mm with widespread small irregular noncalcified nodules in bilateral lung fields and moderate upper lobe predominant centrilobular emphysematous disease (Fig. 1A). He was referred to an interventional pulmonologist, who recommended obtaining sputum cultures, which were acid-fast bacilli (AFB) stain negative, but after 5 weeks, Mycobacterial cultures grew *Mycobacterium shimoidei*.

He was referred to infectious disease and seen in clinic six weeks after culture resulted with improved but persistent intermittent productive cough and dyspnea on exertion. Vital signs were unremarkable except for mild tachycardia. On examination, he was cachectic with bilateral wheezes, but the rest of the exam was unremarkable. Labs about two weeks before the clinic visit showed mild leukocytosis, normal liver enzymes, and renal function. Given the indolent nature of NTM in general and the rarity of *Mycobacterium shimoidei* infection, the decision was made to repeat sputum AFB stains and cultures to help differentiate between possible colonization versus true infection. After three weeks, AFB sputum cultures grew *Mycobacterium shimoidei* again and the isolates were sent to reference laboratory for susceptibility testing*.*

A week later, he was seen in the clinic with unchanged symptoms. He met the microbiological criteria for diagnosis of NTM, with two separate expectorated sputum cultures growing the organism in addition to radiological evidence of cavitary lesion with clinical symptoms suggestive of active infection. Since there are no standardized treatment guidelines for the treatment of *Mycobacterium shimoidei*, empiric antibiotics were chosen based on *M. shimoidei* susceptibilities reported in previous case reports. He was started on a triple-drug regimen consisting of oral azithromycin 500 mg daily, ethambutol 800 mg daily, and rifabutin 300 mg daily.

On clinic follow-up after two months of treatment, the cough had drastically improved but now had decreased appetite, which was attributed to rifabutin; hence, it was stopped. The culture susceptibilities resulted nine weeks after empiric antibiotics were started as seen in Table 1. Based on the sensitivities, moxifloxacin was started, but he developed hot flashes, lightheadedness, and nausea after the first dose, followed by persistent lightheadedness; hence, it was stopped after four days. CT chest about 2.5 months on treatment showed a new anterior left upper lobe cavitary lesion measuring 38.6 mm × 24.0 mm with the old posterior cavitary lesion with volume loss measuring 14 mm × 10.6 mm and the diffuse small noncalcified nodules improved (Fig. 1B). Hence, 600 mg of linezolid once daily was added to the regimen in hopes of better controlling the infection. He developed diarrhea after a few doses and thus was stopped. He remained on two drug regimens, ethambutol and azithromycin. Repeat Mycobacterial cultures after 2.5 months on therapy remained negative.

**Table 1:** *Mycobacterium shimoidei* susceptibilities. EMB -ethambutol, KAN – Kanamycin, AMI- Amikacin, CAP – Capreomycin, RMP- Rifampin, INH- Isoniazid, SM- Streptomycin, ETH- Ethionamide, CIP- Ciprofloxacin, RIF- Rifabutin, CLA- Clarithromycin, CZA- Clofazimine, CYC- Cycloserine, PZA- Pyrazinamide, RFP- Rifampicin, LF- Levofloxacin, LZ- Linezolid, MOX- Moxifloxacin.

Article	Susceptibilities																		
	EMB	KAN	AMI	CAP	RMP	INH	SM	ETH	CIP	RIF	CLA	CZA	CYC	PZA	LF	RFP	LZ	MOX	SMX
Tsukamura 1975	S	S		S	R	R	R	R											
Rusch-Gerdes 1985	S				R	R	I												
Tortoli 1991	R	S			S	R	S	S	R										
Heller 1996	S		S		R	R	S	S	S	S	S	S		R					
Auregan 1997	S					R	S	R						R		R			
Goudge 1998	S		S			R		S	R	S	S		S	S		R			
Mayall 1999	R	R	R			R		R	R	S	R		R	S		S			
Takayama 2006	S	R				R	R	R					S	S	R	S			
Saito 2007	S	S				R	S	S					S		S	S			
	S	S				R	S	S					S		S	S			
Ohmae 2013	S	S				R	S	S			S				S	S			
Galizzi 2013	S		S		R				R	S	S						S	S	S
Kanajii 2013	S	S				R	S	S					R		S	R			
Nagano 2019	S		S				S				S				S	R			
Acero 2023	S	S		S		R	R	S						R		R			
Present case			S		R			I	I	S	S						S	S	S

On clinic follow up about six months on therapy; patient reported less shortness of breath with decreased cough and sputum production. AFB sputum cultures obtained from this visit remained negative at six weeks. On clinic follow up about 9 months on therapy; CT scan showed stable posterior lesion measuring 13.5 mm × 11.9 mm and anterior lesion had decreased in size measuring 28.7 mm × 14.8 mm as seen in Fig. 1C. He noted having no shortness of breath and minimal cough. He was continued on the two-drug regimen for total duration of 12 months from negative sputum cultures.

## 3 Discussion

*Mycobacterium shimoidei* are Gram-positive acid-fast rods that were first isolated in Japan in 1962 but described first in literature by Tsukamura, Shimoide, and Shaefer in 1975 [4]. They are a part of the nontuberculous mycobacterium (NTM) group and, based on their microbiological characteristics in the lab, are subclassified into slow-growing non-photochromogenic NTM. Historically, NTMs are ubiquitous, found commonly in the soil and natural water bodies such as lakes and equipment such as water heaters and ice machines, but *Mycobacterium shimoidei* has only been isolated in the human respiratory tract thus far [3,5].

We used key words *Mycobacterium shimoidei* in PubMed and google scholar search engine, excluding abstracts without sufficient data, 41 cases of M. shimoidei have been published which met the IDSA criteria for NTM infection [[4], [5], [6], [7], [8], [9], [10], [11], [12], [13], [14], [15], [16], [17], [18], [19], [20], [21], [22], [23], [24], [25], [26], [27], [28]]. Though the cases have been worldwide, only 3 cases have been reported in the US previously. The following papers Tsukamura 1975, Tsukamura 1987, Auregan 1997, Takayama 2006, Saito 2007, Ohmae 2013 were translated using google image translator. Ohmae 2013 contained a record of two previous cases from previous published cases by Yamamoto and Kodera. Baird 2017 was a compilation of cases in Australia but only cases with active symptoms were included. All three cases in the US previously reported were poster presentations.

The average age of the cases was 64.5 years and 65.5 % were males [[4], [5], [6], [7], [8], [9], [10], [11], [12], [13], [14], [15], [16], [17], [18], [19], [20], [21], [22], [23], [24], [25], [26], [27], [28]]. Most patients had preexisting lung diseases such as, COPD or interstitial lung disease, previous MTB or other NTM lung infection, and heavy smoking history. Commonly presented with fever, cough, dyspnea, weight loss, fatigue, and sometimes hemoptysis. Cavitary lung lesions were most seen about 73.6 %, followed by nodular consolidations then as lung masses. Only 15 of the previous cases reported susceptibilities for *M. shimoidei* which have been summarized in Table 1 with the addition of our case. Susceptibilities of isolates as follows; 100 % to rifabutin, 87.5 % to ethambutol, 85.7 % to clarithromycin, 83.3 % to amikacin, 61.5 % to ethionamide, but all isolates were resistant to isoniazid and 50 % to pyrazinamide, 84 % to rifampin. Due to the rarity of the disease, there had been a paucity of data available hence management was widely varied but in most cases a combination of three antibiotics. The duration of treatment varied as well but, in most cases, at least 12 months or longer was used. The mortality rate was 25 % but this could be artificially elevated given that some could have died from co-morbid conditions. The findings of the literature review are summarized in Table 2 [[4], [5], [6], [7], [8], [9], [10], [11], [12], [13], [14], [15], [16], [17], [18], [19], [20], [21], [22], [23], [24], [25], [26], [27], [28]].

**Table 2:** Summary of the previously reported cases of *Mycobacterium Shimoidei.* AZA – azithromycin, EMB – Ethambutol, KAN – Kanamycin, AMI – Amikacin, CAP- Capreomycin, RMP – rifampin, INH – Isoniazid, SM – Streptomycin, ETH – ethionamide, CIP- ciprofloxacin, RIF – Rifabutin, CLA- clarithromycin, CZA – Clofazimine, CYC – Cycloserine, PZA – pyrazinamide, RFP – rifampicin, LF- Levofloxacin, LZ – Linezolid, MOX- Moxifloxacin.

Articles	Age	Sex	Risk factors	Symptoms	Radiology findings	Diagnosis	Management	Outcome	Country
Tsukamura 1975	56	M		none	Cavitary lesions	Sputum	KAN, ETH, EMB – unknown duration	Died	Japan
Rusch-Gerdes 1985	79	M	Silicosis with cavitary lung lesions	Fever, cough, hemoptysis,	Cavitary lesions	Sputum	INH, protionamide, RFP, switched to SM and isoprodian for 6 weeks	Survived	Germany
Tortoli 1991	84	M	Asthma, emphysema, hard rock miner,	Cor-pulmonale	Pleural calcifications, left supra hilar density,	Sputum		Died	Canada
Chomyc 1990	68	M	Previous TB infection (>30 yrs prior)	TB attack?	Cavitary lesions	Sputum	INH, SM, EMB, KAN, RMP, p-aminosalicyclic acid for 4 months	Died	Italy
Miller 1991	65	M	Heavy smoker for 42 years,	Fever,	Left upper lobe mass and right sided upper lobe air space disease	BAL	Antibiotics −eft upper lobe resection –path was SC Ca	Survived	Canada
Heller 1996	48	F	MTB at age 37 years old treated since mother died from MTB	Asymptomatic	Right apex cavitary lesion with disseminated bilateral nodules	BAL	RFP, PZA, and INH for 4 months then switched to SM, EMB, CLA, RIF for 4 months then switched to EMB,CIP, and RIF for 18 months.	Survived	France
Auregan 1997	41	F	Previously treated for MTB with residual right apex cavitary lesion	Hemoptysis, unintentional weight loss, fever, SOB,	Right apex cavitary lesion	Sputum	SM, INH, EMB for 1 month, then RMP, INH, EMB for 2 month, then INH, EMB for 9 months. No treatment for 15 months then SM, EMB, RMP, INH and PZA for 2 weeks	Died	Madagascar
Goudge 1998	75	M	Heavy smoker for 50 yrs, bullous emphysema, recurrent pneumonia, abscess, infected lung cysts, history of left upper lobectomy, chronic extrinsic allergic alveolitis	Malaise, anorexia, night sweats, weight loss, increasing dyspnea and productive cough,	Left apex opacification with central cavitary lesion	Sputum	EMB, PZA, CLA, for 1 month with side effects hence switched to PZA, CLA, RIF, then cefotaxime was added. Hospice due to poor nutritional status	Died	Australia
Mayall 1999	53	F	Heavy smoker for 35 years, her mother had MTB, previous pneumothorax, extensive pulmonary fibrosis	Progressive fatigue, weight loss, generalized edema, painless dysphagia, productive cough	Left apex cavity, apical pleural thickening	Sputum	INH, RMP, PZA, EMB for 10 days – died after being on therapy for 10 days	Died	Australia
Sundman 2000	59	F	COPD, emphysema, smoker,	Fever, chest pain, increased dyspnea for 5 weeks	Left cavitary lesion	Sputum	CIP and clindamycin for 4 days then metronidazole and trovafloxacin for 6 weeks.	Survived	Sweden
Koukila-Kahkola 2000	78	F		Cough		Sputum	Observed	Survived	Finland

Takayama 2006	68	M	Previous Heavy smoker,	Fever, cough		Sputum	RMP, EMB, PZA, CLA for 8 weeks then PZA switched to CIP for total 6 months.	Survived	Japan
Saito 2007	45	M	Previous treated for MTB for 6 months,		Left upper lung cavitary lesion.	Sputum	INH and RFP	Unknown	Japan
	75	M	Previous treated for MTB for 6 months,	Fever, cough, weight loss, generalized fatigue		Sputum	RFP, EMB, SM, CLA – SM stopped after 3 months and EMB stopped after 10 months due to decreased vision and RFP and CLA completed 11 months therapy	Survived	Japan
Ohmae 2013	54	M		None	Cavitary with mass		Surgery	Improved	Japan
	75	F	Interstitial pneumonia,	Fever and cough	Micronodular opacities		RFP, EMB, CLA	Improved	Japan
	77	M	Smoker,	Cough	Cavitary and nodular lesions	Sputum	CLA, RFP, EMB for one month then CLA switched to AZA continued for 3 months	Improved	Japan
Galizzi 2013	53	F	History of MTB in her childhood – 40 yrs prior	Dry cough, dyspnea on exertion, recurrent sore throat,	Peri-bronchial consolidations w/ tree in bud	BAL	EMB, AMI, RIF, CLA for one month, RIF stopped after one month, AMI for 3 months, CLA and EMB was continued for 18 months	Survived	Italy
Kanaji 2013	83	M	Previous heavy smoker,	Productive cough, proteinuria, renal impairment	multiple cavities in bilateral lung fields	Renal biopsy	EMB, CLA, RFP, for total 18 months	Survived	Japan
Trivedi 2016	64	F	Emphysema, 40 PPD smoker quit 4 months,	Hemoptysis	Cavitary lesions	2 sputum cultures	EMB, RMP, CLA for 2 months then RMP switched to RIF and AMI was added for 3 months.	Improved at 9 month follow up	USA
Baird 2017	60	M	COPD, asthma	Productive cough and weight loss	Cavitary lesion and nodules	Sputum and BAL x4	Observed	Stable /survived	Australia
	75	F	COPD,	Productive cough, weight loss,	Cavitary lesions and nodules	Sputum x1	None	Died	Australia
	72	M	COPD, bronchiectasis,	Cough, dyspnea, weight loss,	Cavitary lesions and nodules	Sputum x3	Observed	Died	Australia
	62	F		Cough, weight loss, night sweats	Cavitary lesion	Lung tissue x1	INH, RMP, PZA, EMB for 6 months	Stable/survived	Australia
	68	M	COPD,	Productive cough, weight loss, hemoptysis, fatigue	Cavitary lesions and consolidation	Sputum x2	CLA, MOX, SMX, for 12 months	Survived/improved	Australia
	70	M	Lung cancer, COPD, bronchiectasis	Productive cough and chest pain	Cavitary lesions	Sputum and BAL x4	CLA, RMP, EMB, for 12 months	Died	Australia

Baird 2017	77	F	COPD	Cough, weight loss, fatigue	Cavitary lesions and nodules	Lung tissue x1	CLA, RMP, EMB for 18 months	Improved	Australia
	68	M	COPD, RA,	Productive cough, weight loss	Cavitary lesion and consolidation	Sputum x3	Observed	Stable	Australia
	76	M	COPD,	Dyspnea, weight loss	Nodules	BALx1	None	Not reported	Australia
	84	M	Lung cancer,	Productive cough	Mass and effusion	BAL x1	Observed	Died	Australia
	84	M	COPD, bronchiectasis	Cough, dyspnea, Fatigue	Consolidation	Sputum X1	Observed	Improved	Australia
	29	M	Cystic fibrosis and bronchiectasis	Cough, dyspnea, weight loss	Nodules	Sputum	AMI, CIP, AZA, CZA for 24 months	Improved	Australia
	74	F	Bronchiectasis	Productive cough	Nodules and consolidation	Sputum	Observed	Improved	Australia
	84	F	Bronchiectasis	Productive cough, hemoptysis, weight loss	Nodules	Sputum	CLA for 2 months	Improved	Australia
Popovic 2017	67	F	Asthma	Dyspnea, productive cough and malaise	Right apex cavitary lung lesion	Sputum	RMP, INH, EMB, PZA for one month then stopped for 1 month then SM, RFP, EMB, CIP, CLA but CIP stopped after 6 weeks but rest continued for 14 months.	Improved	Croatia
Braganza- Menezes 2018	69	M	Asbestos related pleural plaque, COPD, heavy smoker,	Productive cough,	Nodules and cavitary lesion	Nodule biopsy, sputum	EMB, AZA, RIF for 2 months – stopped to treatment intolerance	Improved	UK
Shin 2018	52	M	Previous MTB, heavy smoker, MAC lung disease treated	Chronic productive cough	Left apex cavitary lung lesion	Sputum	AZA, EMB, RFP, for 15 months	Improved	Korea
Nagano 2019	61	M	COPD, RA, smoker, on prednisone (7.5 mg/day)	Cough, hemoptysis,	Right apex cavitary lung lesion	Sputum	CLA, EMB, LF, for 18 months	Improved	Japan
Shende 2022	35	M	Previous M. Kansaii lung infection treated, silicosis	Cough and weight loss	Cavitary lesions	Sputum			USA

Acero 2023	45	M	Smoker	Night sweats, weight loss, productive cough	Bilateral apex cavitary lesions with nodules	Sputum	Rifabutin, EMB, CLA for 17 months	Improved	Spain
Majoub 2024	72	F	RA, diffuse emphysema,	Dyspnea, cough,	Cavitary lesions	BAL	RIF, EMB, CLA for 18 months	Improved	USA

In our patient, given the numerous side effects of different antibiotics, he has been treated largely with ethambutol and azithromycin, on which he has improved clinically and the cavitary lesions have decreased in size. As per IDSA NTM treatment recommendations, we will continue current therapy for 12 months from negative sputum cultures.

## 4 Conclusion

We present the fourth case of *Mycobacterium shimoidei* infection in the US, in a 72-year-old man who presented with cough, dyspnea, and weight loss. He was treated empirically with oral azithromycin 500 mg daily, ethambutol 800 mg daily, and rifabutin 300 mg daily but due to medication side effects rifabutin was stopped and other antibiotics were attempted based on susceptibilities, but he was unable to tolerate them. Eventually he treated with only ethambutol and azithromycin on which he improved clinically and cavitary lung lesions decreased in size.

## Author contributions

The authors confirm contributions to the paper as follows: clinical case identification – D.I, draft manuscript preparation, data collection − S.G., analysis and interpretation of results S.G., manuscript editing D.I., S.G. All authors reviewed the results and approved the final version of the manuscript.

## Patient consent

Patient consent was obtained, and anonymization of the case was done.

## Financial support

This study was not funded.

## CRediT authorship contribution statement

**Siddartha Guru:** Writing – review & editing, Writing – original draft. **David Ingram:** Writing – review & editing, Supervision.

## Declaration of competing interest

The authors declare that they have no known competing financial interests or personal relationships that could have appeared to influence the work reported in this paper.
